# Walking Football Programme for Elderly People: Study Protocol

**DOI:** 10.3390/sports13050149

**Published:** 2025-05-17

**Authors:** Jofre Pisà-Canyelles, María Mendoza-Muñoz, Jesús Siquier-Coll, Jorge Pérez-Gómez

**Affiliations:** 1 Health, Economy, Motricity and Education (HEME) Research Group, Faculty of Sport Sciences, University of Extremadura, 10003 Cáceres, Spain; 2 Promoting a Healthy Society (PHeSO) Research Group, Faculty of Sport Sciences, University of Extremadura, 10003 Cáceres, Spain; 3 IM-PEPH (Improving Physical Education, Performance, and Health), Department of Communication and Education, Universidad Loyola Andalucía, 41704 Sevilla, Spain

**Keywords:** adapted sport, health, inclusion, older people, physical activity

## Abstract

This study aims to evaluate the effects of a Walking Football (WF) programme on physical fitness, body composition, health-related quality of life (QoL), and happiness in men and women over 60 years. WF is proposed as a safe and accessible activity to counteract physical inactivity and chronic conditions in ageing populations. A randomized controlled trial will be conducted with 120 participants (both sexes), assigned to either an experimental group (WF intervention) or a wait-list control group. The six-month intervention includes bi-weekly sessions with skills training, match simulations, and fitness exercises. Assessments will be performed at baseline, 3 months, and 6 months. Primary outcome measures will be: body composition (weight, BMI, fat and lean mass), muscular strength, aerobic endurance, agility, range of motion, and subjective well-being (QoL and happiness scales). Linear mixed-effects models and ANCOVA will be used to analyse the data. It is expected that WF will lead to improvements in physical and mental health, contributing to active ageing. This study may also support WF as a tool for social inclusion and well-being in older adults.

## 1. Introduction

The average age of the global population is getting higher [1]. As a consequence, ageing can lead to problems of health, social and emotional well-being, as there is an increased incidence of cardiovascular diseases, metabolic and cognitive disorders [2], loss of both muscle mass and muscle quality [3,4] as well as bone mass [3,5], and chronic inflammation [6]. In the coming years, the number of older people is expected to increase, which will lead to a higher demand for health services [7]. This increase poses fiscal and social challenges for governments, especially as older people are more likely to suffer from chronic diseases compared to the younger population [1]. In addition, happiness is crucial for health and QoL, especially among older adults who often face significant life changes that can impact their well-being [8]. Older adults often experience significant life changes, such as losing a spouse, health issues, isolation, and a loss of purpose, which can greatly impact their happiness [8].

In general, in the case of Western societies, there are difficulties in promoting active and healthy ageing [7]. The social complication lies in the physical activity (PA) levels of this population. As people age, the frequency of participation in regular PA decreases [9,10,11] and thus increases levels of physical inactivity and sedentary lifestyles, defined as sitting or lying down for prolonged periods of time [12,13]. Adults mainly do not meet the recommended levels of PA [9,11,14,15]. Such inactivity, coupled with the diseases of old age, increases the likelihood of developing chronic non-communicable diseases [16], leading to a decline in mental health [9] and increasing the risk of mortality from any cause [10]. The management of the quality of life (QoL) of this population is a complex and difficult problem to address due to its multitude of possible factors. Therefore, it is essential to promote PA to maintain a good QoL. PA and exercise have been shown to have a positive effect on some of the negative factors of ageing [17,18,19,20].

Related to the common early problems of ageing, PA has been shown to be a key element in reducing the risk of chronic diseases and improving overall health, with physiological benefits [17] and psychological [18], as well as improving memory capacity [21]. Within these psychological benefits, it is important to highlight the positive relationship between PA and happiness [22]. Just 10 min of PA a week can increase happiness levels [23].

In view of the major problem of physical inactivity in this population, there is a need for solutions and/or ideas to increase participation levels. One possible way to encourage the adoption of increased PA is through participation in sporting activities [24,25]. Traditional interventions, such as strength training and multicomponent exercise programs, have demonstrated efficacy in enhancing muscle strength, balance, reducing fall risk, better physical fitness, and ability to perform daily tasks among older adults [26,27]. Nevertheless, such programmes may not invariably engender long-term adherence, due to factors such as diminished engagement and constrained social interaction [28]. It is evident that team-based sports have the potential to serve as a particularly engaging form of PA for older adults [25], given their social and physical benefits [29]. However, many traditional sports pose challenges in this age group due to their intensity and the risk of injury. This has resulted in the creation of adapted versions that maintain the fundamental components of the sport while mitigating risk. An exemplar of this is Walking Football (WF).

In this sense, conventional football is one of the most popular sports in the world [30,31], which could be interesting to develop in this population to increase PA levels. In the literature, we can find articles that defend the practice of football as medicine [32,33]. It is fun, social and improves metabolic, cardiovascular and musculoskeletal fitness in different age populations [33,34,35], in addition to preventing falls and fractures [34]. Unfortunately, in older adults, the physical demands of playing some sports such as football can be an obstacle to participation. Thus, adapted sports, which modify various aspects of the conventional sporting structure in order to facilitate the participation of any person, can play a crucial role [36]. For this reason, in recent years a more accessible form of the sport, called WF, has been developed [37]. Its origins date back to 1932 in England [38]. This sport has gained popularity in Europe [39] and new clubs, teams and competitive leagues have also been established in North America and Southeast Asia [37,39], expanding throughout the world [24].

The WF is perfect for those who wish to participate in a team sport and have the physical ability to perform moderate and/or intense PA, but cannot, should not or do not want to run, among other variations of traditional football rules [39]. The main objective of this adaptation is to create a sporty, safer and therefore more accessible and attractive variant for older people [37], but it is also used for initiation, rehabilitation and/or for people who have mobility limitations [24]. It is mainly gaining popularity in the 50 and older population as a lower intensity and safer sport than traditional football [40,41,42].

The scientific literature on the effect of PA on health is abundant, however, research is more limited in sports and specific types of exercise for the adult population [43]. As it is an emerging sport, research is still scarce and with diverse methodologies [24]. However, early studies of WF demonstrate cardio-vascular health benefits [37,39], bone health [37] and improvements in body composition [40,42,44], as well as psychosocial improvements [45]. The majority of extant studies have focused on male subjects [24], there is only one of exclusively women with an average age of 40 years [39] and the others with less than 50% participation of women over 55 [24]. It is crucial to consider the differences between the sexes in order to achieve a comprehensive understanding of the subject. Preliminary data suggest that although men tend to play for longer durations, both sexes experience comparable internal load demands during WF sessions, indicating similar physiological responses to effort [46]. Furthermore, participants of both sexes have highlighted WF as a beneficial activity for maintaining physical and cognitive health, fostering social connections, and reinforcing a sense of community and mutual support [47]. However, no study compares the differences between women and men in relation to QoL and happiness.

Therefore, the aim of this study is to present a research protocol to evaluate the effects of a WF programme on fitness, body composition, health-related QoL and happiness in people over 60 years of age.

## 2. Materials and Methods

### 2.1. Study Design

**Figure 1:**
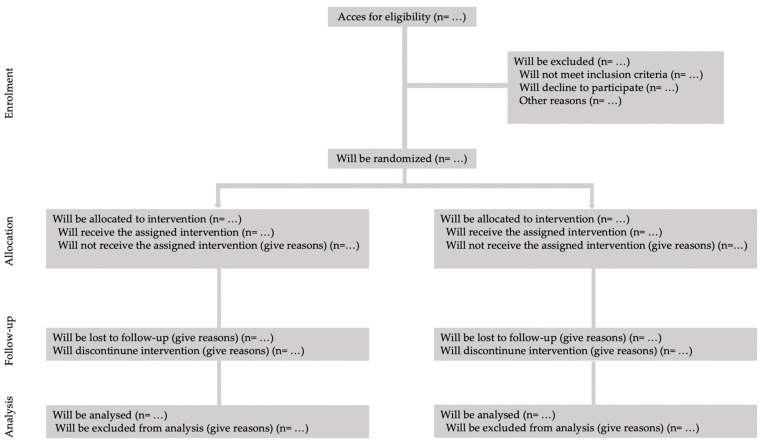
Consort flow gram.

This study will adhere to the CONSORT (Consolidated Standards of Reporting Trials) guidelines for randomized controlled trials, ensuring methodological rigor and transparency throughout the research process [48]. The design will consist of a randomized controlled trial featuring a 6-month intervention phase. Participants will be randomly assigned to either the intervention group, which will engage in the WF program, or a group that will not receive the intervention (Figure 1). Assessments will be conducted at three key time points: baseline (prior to the intervention), mid-intervention (at three months), and post-intervention (at the end of the 6-month program). This structured approach will facilitate a comprehensive evaluation of the intervention’s effectiveness, allowing for the analysis of changes in health metrics and overall well-being over time. By employing this rigorous design, the study aims to provide robust evidence regarding the impact of WF on the targeted population.

### 2.2. Ethics Approval

Ethical approval for this study was obtained from the Bioethics and Biosafety Committee of the University of Extremadura (149/2022). This ensures that the research adheres to the highest ethical standards, prioritizing the welfare and rights of all participants involved. In addition to institutional approval, the study protocol has been registered with the Australian New Zealand Clinical Trials Registry (ANZCTR) under the registration number ACTRN12624000149561. This registration serves to enhance transparency and accountability in the research process, allowing for public access to the study’s details and methodology. The registration can be accessed at https://www.anzctr.org.au/Default.aspx (accessed on 17 October 2024). By securing both ethical approval and trial registration, the study aims to uphold the principles of ethical research conduct, ensuring that participant safety and informed consent are prioritized throughout the duration of the trial. This commitment to ethical standards is essential for fostering trust and integrity in the research community.

### 2.3. Sample Size

To determine the necessary sample size for this study, an a priori power analysis was performed using G*Power^TM^ software 3.1. [www.gpower.hhu.de (accessed on 20 January 2025)]. The analysis was based on a mixed ANOVA model (within-between interaction), which is commonly used in studies with repeated measures involving multiple groups. Using an alpha level (α) of 0.05 and a desired power (1−β error probability) of 0.80, the analysis revealed that a sample size of 48 participants would be sufficient to detect statistically significant effects. The study will account for an expected loss to follow-up of approximately 25% in the sample size calculation [49]. A total of 120 participants, 60 women and 60 men, will be randomly allocated to either the experimental group (EG) (*n* = 60) or the wait-list control group (CG) (*n* = 60).

### 2.4. Randomisation and Blinding

Participants will be randomly assigned in a 1:1 ratio to either the EG, which will receive the WF training, or the CG, will continue with their usual daily life (Figure 1). To ensure the integrity of the randomization process, a member of the research team who is not directly involved in the trial will generate a simple randomization sequence utilizing Research Randomizer software (version 4.0; accessible at http://www.randomizer.org, last accessed on 31 January 2025) [50].

The details of the group assignments will be securely stored in a password-protected file to maintain confidentiality and prevent any potential bias. Furthermore, during the data analysis phase, researchers will remain blinded to the group allocations, meaning they will not know whether participants belong to the EG or the CG. This blinding process is crucial for minimizing bias and ensuring that the outcomes are evaluated objectively, thereby enhancing the validity of the study’s findings.

### 2.5. Participants

This study will be conducted in the province of Cáceres, located in Extremadura, Spain, targeting individuals aged 60 years and older. The recruitment strategy will primarily focus on engaging various associations and organizations that cater to older adults in Cáceres, including day care centre, clubs, sports associations, neighbourhood groups, universities for seniors, and residential facilities. Additionally, collaboration with the local city council and the municipal sports institute will be sought to enhance outreach efforts. These social and institutional entities will assist in disseminating promotional materials, including posters and an online or printed questionnaire designed to formalize contact and gather essential information from interested participants.

The questionnaire will collect pertinent details such as the participant’s name, surname, age, availability, and contact number. Furthermore, all participants will be required to read and sign an informed consent document that outlines the study’s objectives, anticipated outcomes, and potential effects, thereby formalizing their agreement to participate. Given that this initiative represents the first of its kind in Extremadura, it is crucial to garner support from a wide array of organizations to ensure effective promotion and regulation of the activity.

To qualify for participation, individuals must meet specific inclusion criteria: (1) to be over 60 years of age; (2) to live in the region of Extremadura; (3) without any contraindication to perform exercise such as walking at moderate or high intensity (i.e., physical activity exceeding 2 METs); (4) must not be using any medication or undergoing treatments that could interfere with or negatively affect the outcomes of the physical exercise interventions (i.e., beta-blockers, diuretics, antipsychotics, statins, or insulin); (5) intention to continue living in the same place, not to move to another city; (6) to be able to be self-sufficient and to be able to communicate (7) to sign the informed consent.

### 2.6. Health and Safety Protocol

To safeguard the well-being of participants throughout the study, a comprehensive set of risk prevention and control measures will be established, as outlined below. Initially, each participant will undergo a thorough assessment conducted by a qualified physician professional prior to commencing the sports program. This evaluation will include a detailed medical history, a physical examination, and essential tests aimed at identifying any potential cardiovascular, respiratory, or musculoskeletal conditions that could potentially constitute a risk during physical activities.

During each session, constant supervision will be maintained. A certified sports science will lead the group, supported by an assistant trained in first aid. Continuous communication with participants will be prioritized to monitor their well-being, with particular attention given to any signs of fatigue or discomfort that may arise.

In terms of emergency protocols, well-defined procedures will be established to address potential incidents, such as falls, injuries, or cardiovascular distress. The team will be equipped with a first aid kit and will have a pre-established plan for promptly contacting emergency medical services when necessary. Regular breaks will be incorporated into the physical activities to prevent overload and excessive fatigue. Special emphasis will be placed on hydration and appropriate attire. Hydration breaks will be scheduled, and participants will be advised to wear suitable clothing and footwear for the activities. They will also be encouraged to refrain from exercising in extreme heat (above 30 °C) or when experiencing fatigue.

Before each session, brief educational talks will be conducted to raise awareness about the importance of listening to one’s body, recognizing signs of exhaustion or pain, and understanding the necessity of halting activity in the presence of discomfort. Additionally, an incident reporting and tracking system will be implemented to ensure that any incidents or injuries are documented and promptly communicated to the study managers. Participants who experience any mishaps will receive follow-up care to assess their health status and facilitate proper recovery. This proactive approach aims to create a safe and supportive environment for all participants, thereby enhancing the overall effectiveness and integrity of the study.

### 2.7. Intervention

The intervention will take place at the sport city of Cáceres, Extremadura, Spain, utilizing a 40 × 20 m artificial turf indoor court, where it will not be possible to regulate the indoor temperature. The goals will be reduced to 1 square meter, and there will be no designated goalkeeper to enhance the motor engagement of all participants. Conventional footballs will be employed for the activities.

The EG will participate in a WF program over a duration of 6 months, which equates to 24 weeks (Table 1) [51,52]. The program’s content will be structured into two distinct phases, each lasting 3 months [51]. The initial phase will focus on general initiation and adaptation exercises, such as ball movement and passing techniques. This will be followed by a directed phase that emphasizes match simulations, complemented by exercises that facilitate the transfer of skills to competitive scenarios, including field positioning and the development of passing lines. Sessions will be conducted twice a week [40,53,54], each lasting 1 h [44,51,54,55]. The composition of teams will be adjusted based on the number of participants attending each session, accommodating groups of 5, 6, or 7 individuals, in accordance with findings from previous studies [24]. This flexible approach aims to ensure that all participants are actively engaged and can benefit from the program, fostering both PA and social interaction within the framework of WF [37,56].

To effectively manage exercise intensity, the sessions will utilize a perceived exertion ratio based on the OMNI 10-point scale [52,54]. This scale ranges from 0, indicating extremely easy exertion, to 10, representing extremely difficult exertion [57]. This subjective measure allows participants to self-regulate their intensity levels, categorizing exertion as light (3–4 points), moderate (5–6 points), vigorous (7–8 points), and maximum (9–10) [57]. This approach not only enhances individual engagement but also ensures that participants can tailor their efforts according to their personal fitness levels and comfort. Throughout the intervention, the intensity of the sessions will progressively increase in accordance with participants’ physical adaptation (Table 1). This is reflected in the planned OMNI scale scores, which are expected to range from moderate (3–4) to moderate-to-high levels (7–8) in the final stages of the programme. This gradual increase is designed to ensure safety, optimise physical improvements, and promote adherence among older adults.

The structure of the session can be found in Table 2. Each intervention session will commence with a structured warm-up, which is divided into two distinct components. The first component is a general warm-up that emphasizes joint mobility exercises and global movements to prepare the body for PA [51]. The second component is more specific [51], incorporating analytical exercises that are commonly utilized in football, such as lateral displacements and changes of direction. Following the warm-up, participants will engage in a traditional match, which may be supplemented with technical and tactical football exercises tailored to the specific phase of the program being implemented.

Participants will be assigned to teams to ensure a balanced distribution. Efforts will be made to achieve an approximately 50% balance of men and women. A similar distribution will be used among the fastest persons, based on the results of the future tests shown in Table 3 (Brisk Walk test). The session will conclude with a cool-down phase that includes relaxation and range of motion exercises, aimed at facilitating recovery and promoting a return to baseline physiological states [51].

The CG will maintain their usual lifestyle and daily routines throughout the intervention period. They will not engage in any physical exercise program comparable to that undertaken by the EG. After the final phase of the study, participants in the CG will be invited to participate in the WF program, ensuring equitable access to the intervention.

### 2.8. Procedures and Measures

Analysis will be conducted at three key points: prior to the commencement of the intervention, at the three-month mark, and upon completion of the program. A variety of tests will be employed to evaluate the specified variables, as detailed in Table 3.

Participants will wear comfortable and light clothing during the measurement process. Before taking the measurements, individuals will be asked to remove their shoes, socks and any heavy clothing, such as coats, jumpers, jackets, etc. They will also be asked to empty their pockets, remove their belts or any other objects, as well as remove any accessories they are wearing, such as pendants, rings, earrings, among others. Height will be measured with a measuring rod (Tanita Tantois, Tanita Corporation, Tokyo, Japan). Body weight will be measured with a bioimpedance meter (Tanita MC-780 MA, Tanita Corporation, Tokyo, Japan), which will also be used to assess body composition parameters [58].

The tests will be carried out prioritising the safety and health of the participants. If they have any pain or any contraindication to perform any test, before or during the activity, their participation must be cancelled to avoid possible injuries or physical problems. The following is a description of tests to assess physical fitness:

a. Handgrip strength

In order to obtain the strength of the flexor muscles of the metacarpals, a digital dynamometer (TKK 5101 Grip-D; Takey, Tokyo, Japan) will be used. The starting position will be standing, holding the dynamometer with one hand at waist level and keeping it aligned with the forearm [59]. The executing arm shall be at the side of the body without touching the body [59]. The test shall be performed by flexing the fingers of the hand with the maximum possible force and maintaining the position of the dynamometer with respect to the forearm present without flexing, extending or rotating the arm [59], with an intraclass correlation coefficient (ICC) of 0.94 [67,68]. The duration of the test should be short, maximum and voluntary [69], approximately 3 s to avoid fatigue [59,68]. A total of three attempts were performed with each hand, beginning with the right hand [68]. The best result obtained in both tests for each hand shall be selected [68].

b. Lower limbs strength

To acquire a variable of the strength capacity of the lower extremity musculature, the Chair Sit to Stand test of standing upright in a chair, standing up and sitting down for 30 s shall be performed. The number of times a person is able to stand up completely from a seated position, keeping the back straight and feet flat on the floor, without supporting themselves with their arms, in a period of 30 s, will be counted (women ICC = 0.92, men ICC = 0.86) [60].

c. Aerobic endurance

The motor capacity of aerobic endurance will be measured with the 6-Min Walk test, which will consist of measuring the distance that each participant is able to walk, in a time of 6 min in a rectangular circuit of 45.7 metres (50 yards), which represents the perimeter of the rectangle, (women ICC = 0.91, men ICC = 0.97) [60].

d. Walking speed

To measure the motor capacity of displacement speed, the Brisk Walk test will be carried out based on timing the time each participant takes to walk a distance of 30 metres at maximum controlled speed [61,62], (ICC = 0.96) [70]. Two attempts will be performed with a 5-min rest interval in between, and the best result obtained will be used for analysis [71].

e. Range of motion

With the motor capacity of range of motion, the Chair Sit and Reach test will be performed in which participants will start seated with one leg extended and slowly bend down, sliding their hands down the leg until they touch or exceed their toes [63], (women ICC = 0.96, men ICC = 0.92) [60]. The distance will be measured in centimetres and two attempts will be carried out for each leg, recording the best result obtained on each leg.

To increase the value of the physical tests, the assessment of subjective health status is proposed. The tests for the assessment of subjective health condition are described below:

a. 15D

QoL is obtained by means of the questionnaire which consists of 15 Dimensions (Cronbach’s alpha = 0.79). For each dimension, there are 5 response grades that are used to obtain a final measure between 0 and 1, where 0 will represent the worst possible QoL and 1 the best. This questionnaire takes into account preferences and allows for a cost-effectiveness analysis [64].

b. SF-12

A 12-question tool (Cronbach’s alpha > 0.70), which is a shortened version of the SF-36 questionnaire, will be available to measure a person’s overall health and well-being. This tool consists of 8 dimensions (physical function, physical role, bodily pain, general health, vitality, social function, emotional role, mental health) and 2 summary components (physical and mental). The dimensions and components will be rated on a scale from 0 to 100, where 0 represents the worst state of health and 100 the best. This questionnaire will provide a useful index, including the SF-6D [65].

c. Happiness

In order to assess happiness, the General Happiness Scale questionnaire (Cronbach’s alpha = 0.86) will be used, which consists of 4 questions where the possible answers range from 1, which represents the worst happiness, to 7, which is the best state of happiness [66].

### 2.9. Statistical Analysis

Baseline characteristics of participants will be presented as mean (standard deviation) for continuous variables and frequency (percentage) for categorical variables. To assess the effects of the intervention, a linear mixed-effects model will be used, as it allows for the inclusion of repeated measures while accounting for intra-subject correlations. This model will include group (intervention vs. control), time (baseline, mid-intervention, and post-intervention), and their interaction (group × time) as fixed effects. Random intercepts for participants will be included to account for individual variability. The statistical analysis plan is appropriate; however, a brief justification for the chosen models is warranted.

An Analysis of Covariance (ANCOVA) will be conducted to assess the impact of the intervention while adjusting for baseline values and potential confounders such as age and sex. For comparisons between groups at different time points, post hoc tests will be performed with Bonferroni correction to adjust for multiple comparisons. If assumptions of normality and sphericity are violated, appropriate corrections (e.g., Greenhouse-Geisser or Huynh-Feldt) will be applied. To estimate the practical significance of the results, effect sizes will be reported using partial eta-squared (η^2^p) for ANCOVA models and Cohen’s d for pairwise comparisons, along with 95% confidence intervals.

All analyses will be conducted following the intention-to-treat principle, including all randomized participants, and a per-protocol analysis will be performed to assess the effects of adherence to the intervention. Statistical significance will be set at *p* < 0.05, and all analyses will be performed using commercially available software (IBM SPSS Statistics^®^ version 30.0; IBM Corp., Armonk, NY, USA, and Microsoft Excel^®^ version 365; Microsoft Corp., Redmond, WA, USA). The linear mixed-effects model and ANCOVA are preferred over traditional repeated measures ANOVA as it offers greater flexibility in handling missing data and unequal time intervals, and better accommodates the hierarchical structure of the data.

## 3. Discussion

This study pursues innovative objectives from a social and physical health point of view as it aims to ensure healthy living and promote wellbeing for all ages. The findings of this initiative will allow a deeper understanding of the adherence and health benefits of older people, as well as estimating the reduction of the economic cost to the public system through the practice of WF. Furthermore, from a business point of view, the project can offer the applicability of a new sports service to be developed in the commercialisation processes of small and medium-sized companies, thus increasing their competitive advantage in the social and health care sector and can be extrapolated to other public and private sectors.

To our knowledge, this is the first study based on a WF intervention in Extremadura and Spain. Worldwide, there is only one study protocol in the Portuguese diabetic population [52]. Moreover, the existing protocol only contemplates male participation. Nevertheless, the two proposals aim to provide an idea of the applicability of a WF programme to promote PA.

In general, older people who engage in sports reduce their levels of inactivity compared to those who engage in non-institutionalised PA [11,72]. This sport participation helps older people to be aware of their functional limits and improve their opportunities later in life [73,74,75]. Sport can improve the physical [76], mental [2] and social [77] health of older people [43]. It also helps participants feel happy that they have challenged social norms about age-appropriateness and have broken down stereotypes about old age [78]. However, more research is needed on the potential application and benefits of modified sports in older adults [43,79].

Other proposals for WF studies for similar populations can be found in the literature. However, there is no consensus on methodology, study variables and/or regulations (Table 4). For the time being, it improves health-related indices in people over 50 years of age [24,37,40,52,54,80,81,82,83]. Furthermore, as it is a group sport, it helps socialization and its respective benefits such as adherence, fun and the creation of friendships [13,45,84,85,86].

Several studies align with our proposal (Table 4), highlighting the importance of analysing body composition in order to gain a more comprehensive understanding of health and well-being [40,42,55,87,88]. The analysis of body composition is a relevant aspect in the evaluation of the effects of programmes or interventions related to PA and nutrition. In our study, we used a bioimpedance instrument. It is crucial to recognize that body composition is closely linked to essential health parameters [89]. In addition to its importance in sports and fitness, a higher percentage of lean mass can decrease the risk of metabolic syndrome [90], bone mass loss [91] and various complications related to sarcopenia [92].

Firstly, it is important to note that only two previous WF studies (Table 4) have used the same bioimpedance instruments as we have [42,87]. One study, conducted during the Ramadan period, showed a significant improvement in body mass index (BMI), which supports our proposal and suggests that these instruments may be effective in assessing changes in body weight [87]. However, this loss of fat mass may be due to the Ramadan period, but on the positive side the WF group increased muscle mass. On the other hand, a pilot study found no improvement and even showed an increase in BMI [42], therefore it could be attributed to the low weekly frequency of the programme, one hour, or the lack of implementation of adequate nutritional strategies. Elsewhere, another study did not use the same methodology to measure body composition, analysed anthropometric measurements and found a reduction in fat mass after 12 weeks of WF [40]. This study provided data on BMI, fat-free mass, fat mass and percentage fat mass, thus extending the information available on the effects of the intervention [40]. In contrast, another pilot study focused only on BMI, without considering body composition in detail, where no differences were found [44]. Three other studies also provided BMI as descriptive data, without analysing the evolution of body composition [39,54,56]. However, although BMI is a useful measure, it is insufficient for accurate epidemiological analyses. Additional methods are suggested to improve the accuracy in describing and predicting the relationship between anthropometric variables and outcomes of interest [93]. All methods of assessing body composition have strengths and limitations [89]. There is no one method that is considered better than another; research must assess the practical needs of its evaluation along with the limitations of each method [94]. Ultimately, these findings are consistent with our proposal to assess, in some way, body composition in relation to WF.

The Tanita bioelectrical impedance system is a valid tool for estimating body fat percentage in older adults and represents a useful and functional alternative for assessments in public health settings [95]. Although there are more accurate methods, such as dual-energy X-ray absorptiometry (DXA) or magnetic resonance imaging, this method was chosen because of its lower cost and practicality for field studies, as it can be easily transported to the measurement site. In addition to its validity, this device allows a quick and complete analysis of multiple variables, which facilitates the evaluation of a larger number of participants in a shorter time.

Secondly, when evaluating the existing literature, we observed that only a few studies agree with our proposed variables studied (Table 4). In relation to forearm strength, only two previous WF studies have used this measure, both of which found no significant differences, supporting that this variable is not very specific to this activity [56,88]. However, the assessment of forearm strength provides a reliable indication of various health conditions and adverse health outcomes in older people [96]. Indeed, grip strength should be considered a ‘vital sign’ in the assessment of older adults in clinical settings due to its predictive validity, simplicity, portability and low cost [97]. As an indicator of biological vitality, grip strength has predictive validity for cognitive decline, mobility, functional status and mortality in community-dwelling older populations [98]. However, in addition to physical characteristics, various psychological factors, such as cognition and attitude towards the test, also influence the results, although they are often poorly considered in standard procedures, especially in research that has focused more on young, healthy adults than on older patients [99]. Grip strength testing using a dynamometer is valid and reliable [67,68,100]. It allows maximal efforts to be made in a controlled and safe manner. Adding value to research with a basic instrument.

Cardiorespiratory ability is critical, not only because of its impact on physical performance, but also because of its relationship with cognitive performance and brain health in older adults, as highlighted [101,102,103]. Regular walking, for example, improves cardiorespiratory fitness and helps prevent disability in older adults, even though age-related physical decline is inevitable [104]. In addition, higher fitness status, as measured by VO^2^, has been associated with lower arterial stiffness in both sedentary populations and in older adults with higher training compared to their inactive peers, suggesting that improving aerobic capacity may mitigate age-related arterial stiffening [105]. Regarding cardiorespiratory fitness, we found that only one study used the 6-Minute Walk test in line with our proposal (Table 4), which showed significant improvements in the results [87]. Other researches have used different tests to assess cardiorespiratory fitness. For example, in a pilot study, they used the step test, in which no significant improvements were observed, but a trend was observed after 8 weeks of WF [44]. This low significance could be due to the low frequency of training, one hour per week, or the short duration of the program. However, a study of WF in prostate cancer patients found significant cardiorespiratory improvements, as assessed by a treadmill exercise test [56]. The 6-Minute Walk test, it is valid for assessing aerobic capacity in older adults [60]. The choice of such a test is due to its simplicity, low cost and ability to realistically reflect daily activities. In addition, is especially useful for detecting functional limitations that may not be evident in other tests, providing a direct and practical assessment of physical performance in everyday life [106].

There is only one study on WF in prostate cancer patients that utilized the Chair Sit to Stand test (Table 4), which found significant improvements in the WF group compared to the CG. The study also evaluated the strength of the knee extensor muscles using a force sensor. The WF group showed significant improvements over the CG [56]. Following the line of strength assessment, a WF study proposes to specifically assess the strength of the lumbar musculature [87], this suggestion might be too analytical and requires more material for the assessment. These findings suggest that performance in the chair stand-up test is useful as it reflects both muscle size and physical function in older people [107]. Lower limb power is closely related to measures of strength, endurance, speed and agility; in older adults with sarcopenia, this relationship is even more pronounced. This suggests that power may be a useful independent measure of functional fitness, applicable in clinical settings with time and space limitations [108]. In terms of test validity, there is a moderately high correlation between performance in the chair stand-up test and maximal strength in the leg press [60], which supports the choice of test. In addition, in support of validity, it was found that scores on the chair rise test decreased with age and were lower in participants with low PA compared to highly active people [60].

Regarding of the concept of agility, muscle strength is necessary for physical fitness, but agility is also essential for older adults to perform everyday tasks effectively [26]. They confirm that greater leg strength is associated with better agility and less fear of falling, which may reduce the risk of falls [26,109]. A physical training programme combined with dual-task activities could be a useful strategy in the prevention of falls [26]. As for walking, age has a limited effect on walking speed, with pathologies being mainly responsible for gait disturbances [110]. Regarding agility, there have been no observed studies related to WF. A recent paper proposes to assess the agility with modified agility T-Test [87]. This test presents an appealing option due to its multidirectional component, in contrast to the Brisk Walk test. While brisk walking enhances cardiorespiratory fitness, muscular strength, and body composition, further research is needed to explore its effects on balance, flexibility, muscular endurance, and overall life satisfaction in the elderly [111]. Brisk walking alone does not meet all health, and wellness needs in the elderly. Therefore, future studies should explore the efficacy of combining different types of exercise, and clarify the principles for optimising physical exercise outcomes [111].

Loss of joint mobility is a widely documented phenomenon in the ageing population [112]. On the other hand, it has been observed that functional abilities, such as getting up from a chair or climbing stairs, depend more on the presence of specific pathologies than on age per se [110]. Joint range of motion decreases with age, especially after the age of 80 years, and is more pronounced in men, while women retain a greater range of motion [112]. Furthermore, passive range exceeds active range, suggesting that voluntary range of motion is more affected than passive range [112]. This deterioration is mainly due to biological ageing, although factors such as physical inactivity also play an important role [112,113]. Although the prevalence of mobility problems in older adults is high, studies suggest that an active lifestyle can mitigate these effects by significantly improving range of motion in the lower limbs [114].

The same applies to the assessment of joint mobility, for the time being there are no testing protocols. However, in some proposals for WF programmes, it is specified to serve a few minutes to work on mobility [56,87], activation/warm-up [42,53,56,80,87,88,115], la relaxation [52,53,54,56] or stretching [87]. This variable is also of interest since people near old age with better mobility tend to have a higher QoL [116]. Diseases that reduce mobility, such as arthritis, can significantly affect their well-being [116].

The validity of the Chair Sit and Reach test as a practical and reliable tool for assessing hamstring flexibility in older adults is supported [60]. Furthermore, the test’s ability to discriminate between different age ranges, particularly between 70 and 80 years of age, reinforces its usefulness in detecting aging-related changes [60]. Although more accurate methods exist, such as floor testing, the ease of application and greater accessibility of the chair-based test, especially in populations with functional limitations, make it a suitable option.

Interestingly, when examining similar sports, such as recreational football, we found one study that matched four of our tests. In this study, they also used bioimpedance tests to assess fat percentage, The Chair Sit to Stand test to assess muscular strength and the 6-Minute Walk test to assess cardiovascular endurance. They showed significant improvements in the aforementioned tests, however, they also applied the grip strength test with no improvement. The results of this study support our choices of variables and show improvements in these measures after the intervention.

PA has a positive impact on the mental well-being of older adults, improving their health and QoL [117,118]. Several studies have shown that exercise promotes subjective well-being by keeping older adults active, reducing stress, improving alertness and preventing social isolation [119]. Although these benefits are clear, more research is needed, especially among sedentary older adults, to better understand how PA affects their well-being in older age [117,119].

In the context of QoL, only two studies have investigated whether the WF intervention had a positive impact on QoL in people over 50. The first study focused on prostate cancer patients and used the health-related QoL questionnaire (EORTC-QLQ-C30) but found no significant improvements. In the second study, different questionnaires were used, such as the RAND 36-Item Short Form Health Survey, the Warwick-Edinburgh mental well-being scale and the self-esteem scale [44]. However, improvements in QoL were also not observed after the WF intervention [44]. Discrepancies in the results could be explained, at least in part, by differences in the assessment methods, treatment regimens and training programmes used in each study. Within the framework of analyzing the influence of physical activity on happiness and subjective well-being, it is noteworthy that evidence suggests the total amount of PA has a greater impact than the specific type of exercise [22]. Furthermore, a bidirectional relationship between PA and life satisfaction has been identified, indicating that promoting movement can yield both physical and emotional benefits, while individuals with higher well-being are also more likely to be physically active [22]. In this context, WF emerges as a viable and effective option, particularly for adult and older populations, as it offers an accessible form of moderate exercise that incorporates social and recreational components key factors in the promotion of happiness

As previously discussed, some of the sample sizes reported in earlier studies showed considerable variability, ranging from 10 to 25 participants [24]. This variation in sample size may influence the generalizability of the results obtained, as smaller samples could have a limited representation of the target population. An examination of the participant demographics reveals a notable majority of men [24]. To date, there are only four articles available with female participation [13,39,88,120], one of which is female-only [39], where heart rate and distance travelled were analysed. The others articles with less than 50% participation records the experiences of the activity [13,88,120]. This could be attributed to the fact that most football fans are men [121]. However, the phenomenon of football is increasing women’s interest, as well as their participation [122].

Among the limitations of the present study is the lack of comprehensive research on experiences related to WF participation, which suggests an area of opportunity for future studies. In particular, the social component of such participation could be key to fostering interpersonal interactions and relationships, as has been noted in previous literature [24]. Besides, future studies should include specific instruments to assess the mental or motivational component, an aspect that we consider in our methodology but that deserves more attention to better understand the overall experience of participants. Another limitation is the absence of a detailed analysis of differences in levels of health-related outcomes among different subgroups of the population. Adopting this approach would help identify gaps and target future interventions to prevent chronic diseases such as obesity, thereby optimizing the public health impact of sports activities. Moreover, the study did not include any control or monitoring of participants’ dietary habits, which could influence certain health outcomes. In this particular study, no preliminary data are presented, but only the study protocol to be carried out in the future is detailed. This can be seen as a limitation of the research. Furthermore, it is important to note that this study is applicable to specific conditions of community-dwelling adults over 60 years of age in Spain, and contextual or cultural differences may limit the generalisability of the findings to other countries or populations.

Prospective avenues for exploration in future research include the investigation of the effects of WF across a broader age range, with extended intervention durations and the potential for increased weekly frequencies. Furthermore, it would be beneficial to incorporate a more comprehensive assessment of physiological, psychological and social factors. For instance, the measurement of alterations in body temperature, caloric expenditure, metabolic pathways utilised, blood glucose levels, heart rate, muscle activation, oxygen consumption, distance traversed, accelerations and decelerations, etc, can be utilised as indicators of physiological responses to exercise. It is not possible to incorporate this measurement into the assessment due to two factors. Firstly, there is a lack of access to the necessary equipment. Secondly, the time constraints of the assessment sessions make it impractical to include the measurement. Additionally, it would be intriguing to establish a correlation between these WF responses and more robust and specific psychological and social studies. This multidimensional approach has the potential to significantly enhance our understanding of the benefits of WF, particularly in relation to general well-being, social connectivity, and its role in active ageing.

The added value of this knowledge primarily lies in understanding how WF programs can enhance health, physical condition, and QoL for individuals over 60 years old. This represents a significant advancement for developing new strategies in this area. Therefore, the possible adherence to these programmes will mean a lower cost to the health system, as well as being able to implement this type of service to other people (adolescents, situations of social exclusion, reinsertion programmes, various pathologies, recovery from injuries, initiation, to work on tactical content, etc.) providing a great social and mental impact. In addition to a proposal for an activity, a methodology adapted to this population is proposed to describe and analyse the possible effect of the activity itself.

## 4. Conclusions

This study presents an innovative research protocol designed to evaluate the impact of WF on physical fitness, body composition, health-related QoL and perceived happiness in people over 60 years of age. Through a randomised controlled trial design, the research will provide rigorous evidence on the effects of this sport in promoting active and healthy ageing.

Given the increase in chronic diseases and the reduction of PA in this population, WF is proposed as an accessible and safe alternative, with the potential to improve multiple indicators of well-being. In addition, this study contributes to closing a gap in the literature by including both men and women, allowing for a more equitable analysis of the benefits of the programme.

The findings derived from this work will not only have implications for clinical practice and public health but can also inform the creation of strategies and policies to promote physical exercise in older adults. In particular, the results may help shape community-based health promotion programs and guide local and national public health interventions that seek to improve functional independence, social engagement, and overall quality of life in ageing populations.

Furthermore, the proposed methodological standardisation will serve as a reference for future research on PA-based interventions in this age group, people aged 60 and over living in the community, being physically and functionally independent.

## Figures and Tables

**Table 1:** Example of a weekly schedule for the walking football program.

Months	Phases	Weeks	Tests	OMNI *
1	General	1	Basal	3
		2		3
		3		3
		4		3
2		5		4
		6		4
		7		4
		8		4
3		9		5
		10		5
		11		5
		12	Intermediate	5
4	Guided	13		6
		14		6
		15		6
		16		6
5		17		7
		18		7
		19		7
		20		7
6		21		8
		22		8
		23		8
		24	Final	8

* OMNI: Ratio perceived exertion scale, 0 to 10.

**Table 2:** Structure of a walking football session.

Phase of the Session	Duration (Minutes)
General warm-up	5
Specific warm-up	5
Main part	45
Cooldown	5

**Table 3:** Summary of the test battery.

Type	System	Variables	Test	Reference
Physiologic	Morphology	Body composition	Bioimpedance	[58]
	Morphology	Anthropometry	Height	[58]
Physical	Muscular	MVIC	Hand Grip	[59]
	Muscular	Lower limb strength	Chair Sit to Stand	[60]
	Cardiorespiratory	Aerobic endurance	6 Minute Walk	[60]
	Muscular	Walking speed	Brisk Walk	[61,62]
	Muscular	Range of motion	Chair Seat and Reach	[63]
Questionaries	Physical and mental	Quality of life	15D	[64]
	Physical and mental	Quality of life	SF-12	[65]
	Mental	Mood	Happiness scale	[66]

MVIC: Maximum Voluntary Isometric Contraction.

**Table 4:** Comparative summary of tests already used in the walking football literature.

Type	Variables	Test	Has the Test Been Utilised?	Reference
Physiologic	Body composition	Bioimpedance	Yes	[42,87]
	Anthropometry	Height	Yes	[42,87]
Physical	MVIC	Hand Grip	Yes	[56,87,88]
	Lower limb strength	Chair Sit to Stand	Yes	[56]
	Aerobic endurance	6 Minute Walk	Yes	[87]
	Walking speed	Brisk Walk	Yes	[61,62]
	Range of motion	Chair Seat and Reach	No	-
Questionaries	Quality of life	15D	No	-
	Quality of life	SF-12	No	-
	Mood	Happiness scale	No	-

MVIC: Maximum Voluntary Isometric Contraction.

## Data Availability

Data sharing is not applicable.
